# Molecular structure of soluble vimentin tetramers

**DOI:** 10.1038/s41598-023-34814-4

**Published:** 2023-05-31

**Authors:** Pieter-Jan Vermeire, Anastasia V. Lilina, Hani M. Hashim, Lada Dlabolová, Jan Fiala, Steven Beelen, Zdeněk Kukačka, Jeremy N. Harvey, Petr Novák, Sergei V. Strelkov

**Affiliations:** 1 Laboratory for Biocrystallography, KU Leuven, 3000 Leuven, Belgium; 2 Department of Chemistry, KU Leuven, 3000 Leuven, Belgium; 3 Department of Biochemistry, Charles University, 12800 Prague, Czech Republic; 4 Institute of Microbiology of the Czech Academy of Sciences, 14220 Prague, Czech Republic

**Keywords:** Intermediate filaments, Cytoskeletal proteins, Molecular modelling

## Abstract

Intermediate filaments (IFs) are essential constituents of the metazoan cytoskeleton. A vast family of cytoplasmic IF proteins are capable of self-assembly from soluble tetrameric species into typical 10–12 nm wide filaments. The primary structure of these proteins includes the signature central ‘rod’ domain of ~ 300 residues which forms a dimeric α-helical coiled coil composed of three segments (coil1A, coil1B and coil2) interconnected by non-helical, flexible linkers (L1 and L12). The rod is flanked by flexible terminal head and tail domains. At present, the molecular architecture of mature IFs is only poorly known, limiting our capacity to rationalize the effect of numerous disease-related mutations found in IF proteins. Here we addressed the molecular structure of soluble vimentin tetramers which are formed by two antiparallel, staggered dimers with coil1B domains aligned (A_11_ tetramers). By examining a series of progressive truncations, we show that the presence of the coil1A domain is essential for the tetramer formation. In addition, we employed a novel chemical cross-linking pipeline including isotope labelling to identify intra- and interdimeric cross-links within the tetramer. We conclude that the tetramer is synergistically stabilized by the interactions of the aligned coil1B domains, the interactions between coil1A and the N-terminal portion of coil2, and the electrostatic attraction between the oppositely charged head and rod domains. Our cross-linking data indicate that, starting with a straight A_11_ tetramer, flexibility of linkers L1 and L12 enables ‘backfolding’ of both the coil1A and coil2 domains onto the tetrameric core formed by the coil1B domains. Through additional small-angle X-ray scattering experiments we show that the elongated A_11_ tetramers dominate in low ionic strength solutions, while there is also a significant structural flexibility especially in the terminal domains.

## Introduction

Throughout evolution, living cells have developed the cytoskeleton, a mechanical network providing both stability and dynamics. The cytoskeleton employs filamentous proteins to scaffold the organelles and generate forces for cellular mobility. In higher animals, cytoskeletal filaments are represented by microtubules (MTs, 25 nm in diameter), intermediate filaments (IFs, 10 nm in diameter) and microfilaments (MFs, 7 nm in diameter). In contrast to the polar MTs and MFs, IFs are nonpolar and thus cannot serve for directed cargo transport. Interestingly, single molecule mechanics studies revealed that IFs are extensible up to 250%^1^. At large deformations where both MTs and MFs break, IFs are still resilient and thus hold the cell together^2^.

In humans, over 70 distinct IF proteins are present. In addition to cytoplasmic IFs, a special IF class is represented by nuclear lamins^3,4^. Primary structure alignment of various IF protein classes reveals a conserved domain organization. All of them contain a ‘signature’ central α-helical rod domain which is flanked by two highly variable terminal domains named head and tail. The rod domain is based on the α-helical coiled coil (CC) structure, which drives association of two IF chains into an elongated filamentous dimer. In the past, the atomic structure for the CC part of the dimer could be established using X-ray crystallography^5,6^. The CC consists of three segments (coil1A, coil1B and coil2) which are interconnected by linkers (L1 and L12). Both linkers vary in length and sequence across different IF proteins, and appear to form flexible ‘hinges’ between the more rigid coiled-coil segments^7^. In particular, in invertebrate IFs the linker L12 was shown to allow a sharp kink^8^.

IF assembly is based on specific associations of the elongated elementary dimers in two directions: the lateral (side-by-side) and longitudinal (head-to-tail). In particular, in cytoplasmic IFs two dimers readily associate laterally to form a tetramer. In the past, it was shown that such tetramers can be maintained in low ionic strength buffers of neutral pH (e.g. 2 mM sodium phosphate^9^). An increase of ionic strength can be used to drive further association of tetramers in the lateral direction yielding the short-living intermediates called the ‘unit-length filaments’ (ULFs)^9–11^. In human vimentin, ULFs were reported to contain, on average, 32 monomers^10^. ULF formation happens within a few seconds, followed by a slower longitudinal association of the ULFs and a radial compaction, ultimately resulting in the mature 10–12 nm filaments^11–13^.

Previous cryoelectron microscopy (cryoEM) studies revealed that mature vimentin IFs typically contained four octameric protofibrils with right-handed supertwisting, while considerable structural variability was also observed^14,15^. Further cryoEM observations of several IF classes were reported recently^16–19^. However, preparations of such filaments, both extracted from cells and assembled in vitro, suffer from considerable heterogeneity, which has limited the resolution of these studies. As a result, direct reconstruction of the IF architecture at atomic detail has thus far not been possible.

As an alternative approach, dissecting the intermolecular contacts between the elementary dimers within the assembled IFs using analytical techniques such as chemical cross-linking in particular was attempted in the past. Given the available crystallographic data on elementary dimer, cross-linking studies could in principle provide sufficient information towards reconstructing the IF architecture in the ‘bottom-up’ way^6^. Early on, Steinert and colleagues have presented a cross-linking analysis of several IF types using disulfosuccinimidyl tartrate (DST)^20–23^. They have found out, depending on the conditions, that the elementary dimers can associate with each other in three distinct lateral ways, called A_11_ (when the two dimers are arranged antiparallel with each other with coil1B segments in register), A_22_ (antiparallel dimers with the coil2 segments in register) and A_12_ (antiparallel, in register dimers). These findings, although based on a limited number of cross-links (XLs) which could be obtained at that time, provided for the first theoretical models of the IF architecture^22^. We argue that, given the challenges that the IFs present to the direct atomic resolution structural methods such as X-ray crystallography and cryoEM, chemical cross-linking still represents a vital tool nowadays.

In this study, we focused on the next assembly step beyond the elementary dimer though examining the soluble tetramers formed by human vimentin in low-salt conditions. Initially, using a series of truncation constructs, we established that the proper tetramer formation requires the presence of the coil1A domain. Next, we applied a modern chemical cross-linking pipeline which involved the use of several cross-linkers and mass spectrometry (MS) based identification of the products. We confirm that soluble vimentin tetramers are exclusively based on the A_11_ type alignment of the elementary dimers, and refine the molecular structure of such tetramers. Beyond an elongated (essentially straight) tetramer, there is also a possibility of ‘backfolding’ of the coil1A and coil2 segments onto the A_11_ tetrameric core after a 180° flexing in both linkers L1 and L12. Finally, our small angle X-ray scattering (SAXS) experiments suggest that the tetramers are mostly straight but there is also a significant degree of structural flexibility.

## Results

### Oligomerization of full-length vimentin and its truncated constructs in solution

**Figure 1:**
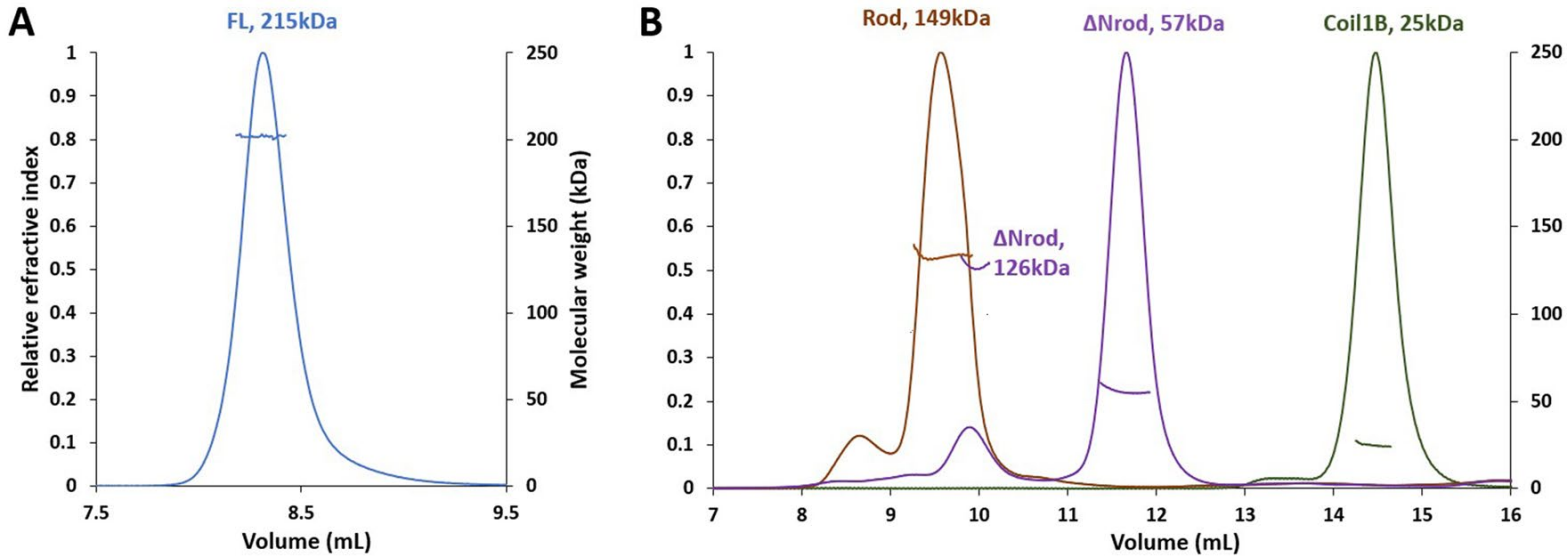
SEC-MALS profiles of vimentin tetramers and its progressive truncations on a Superdex 200 Increase column. Across each elution peaks, MALS-derived molecular weight profiles are also shown (right axis). (**A**) Elution of FL vimentin in LS buffer (2 mM HEPES, pH 8.2). (**B**) Elution of different truncations in HS buffer (10 mM HEPES, 150 mM NaCl, pH 7.5).

Initially, we obtained a full-length (FL) human vimentin sample through recombinant expression in *E. coli*. The protein was purified from inclusion bodies to > 95% purity (as judged by SDS-PAGE with Coomassie staining) in the presence of 8 M urea, and re-natured through a step-wise removal of urea into 2 mM HEPES buffer, pH 8.2 (low-salt conditions, LS). Previously, such dialysis-based procedure was shown to yield soluble tetramers in both 5 mM Tris–HCl (pH8.4) and 2 mM Na phosphate (pH7.5)^9^. However, the Tris buffer could not be used here, since its primary amines react with cross-linkers containing NHS ester groups. Moreover common impurities of phosphates may decrease cross-linking yields with this chemistry (P. Novak, unpublished observations). These considerations led us to choose the HEPES buffer. Under these conditions, the protein eluted as a single peak from a Superdex 200 Increase column (Fig. 1A). SEC-MALS analysis indicated a constant mass of 215 kDa across the elution peak, which exactly corresponds to the theoretical mass of a tetramer.

In order to explore which regions are essential for tetramerization, we also prepared three vimentin constructs with progressive truncations: the rod domain, a yet shorter construct including the coil1B and coil2 domains (ΔNrod), and a coil1B construct (Table 1). The truncated constructs were purified in non-denaturing conditions. Shortly before SEC-MALS analysis, the samples were denatured in 8 M urea to disassemble any complexes or aggregates that may have formed during purification and storage, and then re-natured by a step-wise dialysis into the LS buffer. Next, the samples were subjected to the same ‘salt jump’ procedure as previously used to assemble the FL protein, *i.e.* addition of an equal volume of 2X concentrated high-salt (HS) buffer (20 mM HEPES buffer, pH 7.5, 300 mM NaCl) and incubation at 37 °C for 1 h. Upon this procedure, the FL protein yields 12 nm wide IFs that closely resemble natively assembled IFs^6^.

**Table 1:** Oligomerization properties of vimentin constructs in HS conditions.

Name	Residues	Method	Theoretical monomer mass (kDa)	Experimental mass (kDa)	Peak elution position (mL)	Oligomeric state
Rod	93–404	SEC-MALS	36.9	149 n.d	9.6 8.6	Tetramer Oligomers
		XL + gel				Tetramer
ΔNrod	150–404	SEC-MALS	30.2	57 126	11.6 9.9	Dimer Tetramer
		XL + gel				Dimer
Coil1B	150–249	SEC-MALS	12.1	25	14.4	Dimer
		XL + gel				Dimer

Next, our SEC-MALS measurements in HS conditions (10 mM HEPES buffer, pH 7.5, 150 mM NaCl) indicated that the rod fragment formed tetramers with a small fraction of higher-order oligomers. Possibly, these oligomers could be octamers, since they elute distinctly later (8.6 mL) than the void volume of the column (~ 7.2 mL) (Fig. 1B). Under the same conditions, the ΔNrod construct (which contains both coil1B and coil2) was predominantly a dimer with an additional minor peak corresponding to tetramers, while the coil1B construct was only forming dimers (Table 1).

### Cross-linking studies

Next, we performed chemical cross-linking of both FL vimentin and truncated constructs, using a panel of homobifunctional cross-linkers with varying spacer lengths *i.e.* disuccinimidyl glutarate (DSG), disuccinimidyl dipropionic urea (DSPU) and disuccinimidyl dibutyric urea (DSBU). Their main reactivity is towards N-termini and lysines. The purpose of these studies was dual. First, analysis of the cross-linked samples on reducing SDS-PAGE provided an independent means to assess their oligomerization in addition to the SEC-MALS experiments. At this step, both the protein concentration and the molar excess of the cross-linker over protein were optimized. The aim of optimization was to obtain intense bands of cross-linked oligomers, while having the monomer band still visible and avoiding a smear of over-crosslinked material on the top of the gel. Second, for the FL protein tetramers, full identification of the formed XLs using mass spectrometry (MS) was carried out.

**Figure 2:**
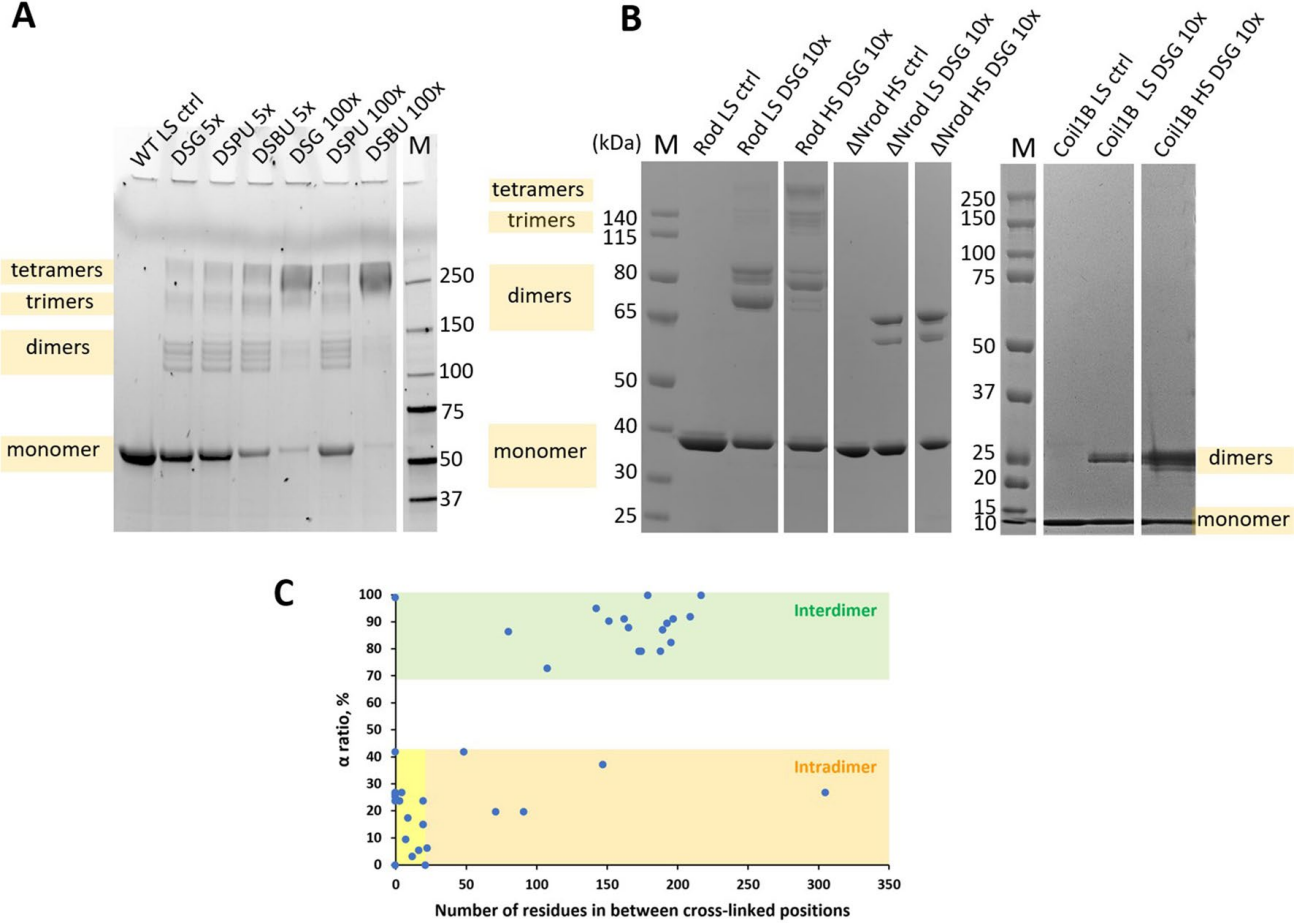
Chemical cross-linking of vimentin samples. (**A**) Reducing SDS-PAGE analysis of the FL vimentin in LS buffer before (control, ctrl) and after chemical cross-linking with DSG, DSPU and DSBU. Molar excess of the cross-linker over the protein is indicated. M stands for a molecular weight marker. The likely composition of different bands is given at the left-hand side of the gels. The original uncropped gels for panels (**A**) and (**B**) are presented in Supplementary Fig. 1. (**B**) Reducing SDS-PAGE of vimentin truncation constructs cross-linked with DSG in either LS or HS conditions. (**C**) Classification of the FL vimentin XLs as intra- or interdimeric using the α-ratio calculated from the ^14^N/^15^N data. The yellow zone corresponds to intradimeric XLs between close-by residues.

For the FL vimentin in LS conditions, the cross-linkers were used in 5 × and 100 × molar excess. Reducing SDS-PAGE revealed multiple cross-linked species, composed of two, three or four protein chains, as judged by the molecular weight standard (Fig. 2A). The original gels are presented in Supplementary Fig. 1. These three species as well as non-cross-linked monomers were present for all cross-linkers and molar excesses. Most observed bands were substructured. This was especially apparent for the dimer where at least four close bands were visible, suggestive of the presence of distinct singly or multiply cross-linked conformations. Notably, no cross-linked species higher than the tetramer were ever observed, pointing to the tetramer being the true soluble species.

Next, truncated vimentin constructs were cross-linked in both LS and HS conditions. Judging by SDS-PAGE, similar results were obtained using DSG (Fig. 2B) and DSBU (Supplementary Fig. 1), even though the latter cross-linker has a longer spacer length. The rod fragment in the HS conditions revealed a clear pattern of dimers, trimers and tetramers, similar to the pattern observed for the FL protein in the LS conditions (Fig. 2A). However, for the rod in the LS conditions, only a small amount of trimers and tetramers was observed. Finally, no cross-linked species beyond dimers were observed for both ΔNrod and coil1B fragments irrespective of the ionic strength used.

### MS analysis of cross-linked FL vimentin

The obtained cross-linked (DSG, DSPU & DSBU at 100 × molar excess) samples of FL vimentin were digested using trypsin and subjected to liquid chromatography and mass spectrometry (LC–MS/MS) analysis. In total, 143 unique XLs were identified (Supplementary Fig. 2A, Supplementary Table 1). Among these XLs, 68 were entirely within the rod domain and 75 involved the head and tail domains.

In order to obtain stronger restraints for three-dimensional modelling, we aimed at distinguishing intra- and interdimeric XLs. This is why we additionally purified a FL vimentin sample with ^15^N isotope labelling. To this end, light (^14^N) and heavy (^15^N) vimentin samples were both prepared with 6.2 M urea. Under these conditions, vimentin only assembles to dimers but not beyond^24^. Both samples were mixed in 1:1 molar ratio and step-wise dialyzed into the LS buffer (see Methods section “^14^N/^15^N cross linking and data processing” for details).

After cross-linking, proteolytic digestion and LC–MS analysis, the data for the ^14^N/^15^N sample were processed by the LinX software^25^. In total, 40 unique XLs within the rod domain were identified (Supplementary Table 2). Several types of cross-linked peptide pairs were possible: both light (L/L), both heavy (H/H) and a mixture of light and heavy (L/H or H/L) (Supplementary Fig. 2B). In the absence of further exchange of monomers across the dimers, the intradimeric XLs would only be represented by the L/L or H/H peptides, while the interdimeric XLs would be represented by all four pairs of peptides: L/L, H/H, L/H or H/L. Accordingly, for each XL, the so-called α-ratio was calculated using the intensities of the four peptide types (see Methods). The theoretical value of this ratio is 1 for interdimeric XLs and 0 for intradimeric XLs. Plotting the α-ratio for all XLs against the distance between the cross-linked positions in the amino acid sequence (Fig. 2C) indeed revealed a good separation between intra- and interdimeric XLs. This ultimately confirmed our initial expectation that the light and heavy dimers, once assembled separately in 6 M urea, are stable and do not undergo any further exchange of monomers.

Next, we have considered all XLs that connected positions less than 20 residues apart. Of the total of 18 such XLs, all were intradimeric (yellow zone in Fig. 2C) except for a single XL, K188–K188, which was interdimeric. Since intradimeric XLs between close-by residues in sequence can be trivially explained in a parallel, in-register CC dimer, they were excluded from further three-dimensional modelling.

This left us with 23 XLs within the rod domain with a clear intra- or interdimer assignment, including 22 XLs between distant residue numbers (> 20 apart) and the K188–K188 XL (Table 2). All these XLs had also been detected in the initial experiment with the light-only protein (Supplementary Table 1). These 23 rod-only XLs became the basis of the three-dimensional modelling of the tetramer (see next section). Of note, the vast majority of the XLs (17 of 23) are interdimeric.

**Table 2:** Identified XLs within the rod domain.

Segments (# XLs)	Residue 1	Residue 2	Cross-linker(s)	Type XL	Elongated model, Cα-Cα dist. (Å)	Compact model, Cα-Cα dist. (Å)
C1B–C1B (2)	K143	K223	DSG, DSS,*,**	inter	**29**	**32**
	K188	K188	DSG, DSS, DSPU, DSBU,*	inter	**16**	**16**
C1A–C1B (1)	K120	K168	DSS	intra	*69*	**34**
C1B–C2 (4)	K143	K294	DSS	inter	**35**	**30**
	K188	K334	DSBU	intra	*169*	**23**
	K223	K294	DSG, DSS, DSBU	intra	*61*	**28**
	K223	K313	DSG	intra	*88*	**31**
C2–C2 (1)	K294	K402	DSBU,*,**	inter	*369*	*62*
C1A–C2 (15)	K97	K120	DSS, DSBU	intra	**33**	**33**
	K97	K292	DSG	inter	**39**	**25**
	K97	K294	DSG, DSS, DSBU	inter	**36**	**25**
	K97	K313	DSS	inter	**32**	**24**
	K97	K402	DSBU	intra	*404*	*85*
	K104	K282	DSS,*,**	inter	**43**	**33**
	K104	K292	DSS	inter	**30**	**21**
	K104	K294	DSS	inter	**27**	**19**
	K104	K313	DSG, DSS	inter	**16**	**19**
	K120	K262	DSG,*,**	inter	**28**	**39**
	K120	K282	DSG, DSS, DSBU	inter	**22**	**17**
	K120	K292	DSS, DSBU	inter	**13**	**14**
	K120	K294	DSS, DSBU	inter	**15**	**15**
	K120	K313	DSS, DSBU	inter	**31**	**36**
	K129	K294	DSG, DSS	inter	**16**	**28**
(23 XLs)					(17 satisfied)	(21 satisfied)

Asterisks indicate same or nearby cross-linked positions as reported by Steinert’s group for DST in^23^ (*) and^9^ (**). The last two columns indicate the distances between the corresponding residues in each of the models. The distances which are compatible with XL formation are in bold. The distances which exceed the cut-off value are in italics.

Finally, to enable the addition of the head and tail domains at later stages of the modelling, we tabulated all XLs made by these domains (Table 3). Here, all XLs found in the initial experiment using the light-only vimentin sample were considered, including the reactivity of cross-linkers not only to amino groups but also to Ser, Thr and Tyr residues. This yielded numerous cross-links for the head domain, even though this domain does not include any lysine residues.

**Table 3:** Identified XLs for the terminal domains.

**Head–Head (1)** S43–T63
**Head–Rod (41)** **S10–K120, S10–K129, S10–K139, S29–K104, Y30–K139, S34–K120, S42–K120, Y61–K120, Y61–K129, Y61–K139, Y61–K143,** *T63–T99,* **S66–K120, S72–K120, S72–K143, S73–K139, S10–K188,** *S25–K236,* **S29–K188, Y30–K168,** *T32–K235,* **S34–K223, T35–K188, S42–K188, S42–K236, S47–K223,** S49–K188, **S55–K235, S55–K236, Y61–K223,** T63–K188, S10–K373, *S29–K313,* **S29–K373,Y61–K282, Y61–K292, Y61–K294,** *Y61–K313,* **T63–K294,** *T63–T317*
**Head–Tail (5)** S10–S409, *S10–K445,* T33–K445, *T37–K445,* Y61–K445
**Tail–Tail (10)** **S419–K445, S420–K445, K439–K439, K439–S459, T441–K439, K445–S438, K445–K439, K445–K445, K445–S459, T458–K439**
**Tail–Rod (12)** **S420–K402,** *K439–K143, K439–T165,* **K439–K402,** K445–K120, *K445–K294,* K445–Y319, **K445–K373, K445–K402, K445–S412, T449–S409, S459–K402**

**Figure 3:**
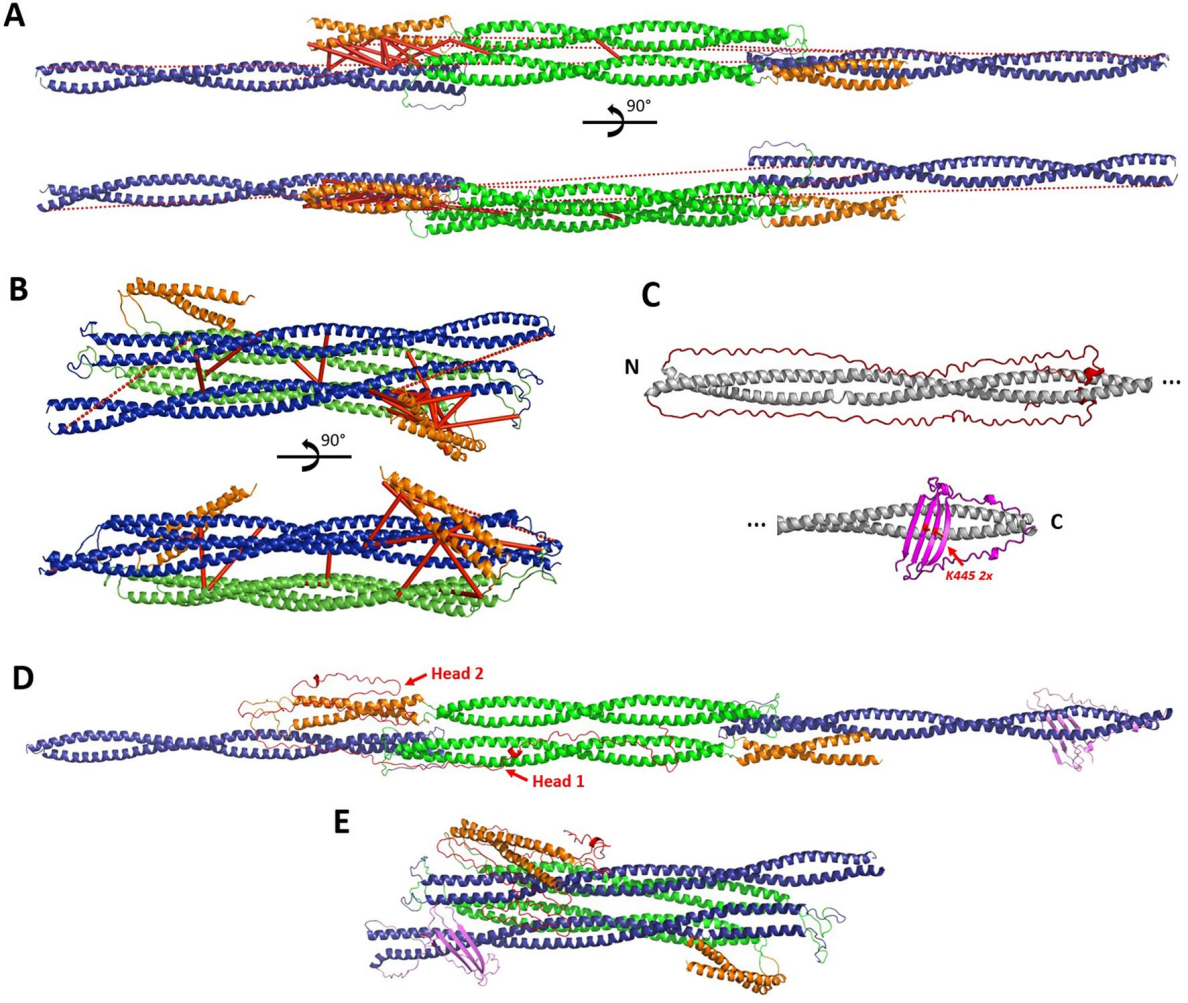
Vimentin tetramer models. (**A**) Elongated A_11_ tetramer. Coil1A is shown in orange, coil1B in green, coil2 in blue. Satisfied XLs are shown as solid red lines and the XLs exceeding the distance cut-offs are shown as red dashed lines. For clarity, only one instance of each unique XL is drawn (for instance, the XL connecting residue K143 in dimer1 and residue K223 in dimer2 is present, while the XL connecting K223 in dimer1 and K143 in dimer2 is not shown). (**B**) Backfolded, compact tetramer. (**C**) AlphaFold modelling of the terminal domains^31^. The upper part shows the head in red together with the beginning of the rod domain (coil1A and part of coil1B) in grey. The lower part shows the tail in magenta together with the end of the rod domain (part of coil2). N- and C-termini of the rod are labelled. (**D**) Elongated tetramer with head and tail domains. For clarity, only the terminal domains for a single dimer are displayed. Here, one of the head domains (Head1) is modelled to mainly interact with coil1B, while the other one (Head2) is mainly interacting with coil1A. (**E**) Compact tetramer model with terminal domains.

The number between brackets is the total number of XLs per domain. Cross-links which are satisfied in the elongated model (Fig. 3A) are in bold. Cross-links which are additionally satisfied in the compact model (Fig. 3B) are Italic.

### Use of isotope labeling and cross-linking to explore tetramer stability

As explained, the use of ^14^N and ^15^N labeled vimentin dimers that had been assembled separately in 6.2 M urea was an efficient means to distinguish the XLs formed either inside or across the constituent dimers within a soluble tetramer. In addition, we used a similar approach to explore whether there was any exchange of dimers between the assembled tetramers. To this end, light and heavy vimentin samples were assembled separately in LS buffer in the standard way, thus yielding tetramers. Thereafter the samples were mixed 1:1 and cross-linked with DSG and disuccinimidyl suberate (DSS). After MS measurements and analysis with the LinX software, we established that the vast majority of the XLs obtained was only represented by the L/L and H/H peptide pairs (data not shown). Only 5 out of 85 XLs obtained using DSG and 11 out of 78 DSS XLs contained all four pairs of peptides (L/L, H/H, L/H or H/L). However, of those few XLs with all four peptide combinations, none were interdimeric based on their quantification. This way we could indeed confirm that in LS buffer vimentin tetramers are stable, without any significant exchange of dimers or monomers across the tetramers.

### Modelling of the vimentin tetramer from experimental data

Here we employed an atomic model of vimentin rod dimer produced using our own CCFold algorithm^26^. To create the starting model of the tetramer, two such dimers were aligned in antiparallel fashion to match the A_11_ type arrangement seen in the crystal structure of the tetrameric coil1B fragment (PDB code 3UF1)^27^*.* Next, the 23 intra- or interdimeric XLs which lay entirely within the rod domains (from the ^14^N/^15^N experiments, Table 2) were used to guide further model refinement.

Our initial modelling procedure involved the optimization of two coil2 segments relative to the central core of the tetramer formed by the coil1B overlap. This procedure was possible due to the relatively long length of the L12 linkers (20 residues). Here, we preserved the overall elongated character of the starting tetramer model by retaining the original orientation of all CC segments. As the result of this optimization, 17 XLs could be satisfied (Fig. 3A). These included both interdimeric XLs occurring between the coil1B domains as well as most XLs (14 of total 15) detected between coil1A and coil2. In our model, the linker L12 accommodates a ‘z’ shape, resulting in an overall reduction of the rod length. Previously, such 'z-folded' conformation was suggested for the nuclear lamin A dimer, based on chemical cross-linking data^28^.

The produced elongated tetramer model (Fig. 3A) still did not satisfy six cross-linking restraints. Of those, five XLs were intradimeric and only one (K294–K402) was interdimeric (Table 2). Correspondingly, we questioned whether further structural adjustments to this model were possible. To this end, we allowed a free rotation of two coil1A dimers and two coil2 dimers as rigid bodies with respect to the tetrameric core formed by the coil1B domains, which is possible through flexing at both linkers L1 and L12. This modelling was performed using the Integrative Modeling Platform (IMP)^29^. This IMP-based modelling attempted to satisfy as many XLs as possible, whereby the latter were specifically applied as either intra- or interdimeric restraints (Table 2). In addition, we restrained the complete tetramer to have the same two-fold symmetry as present in the antiparallel overlap of coil1B segments^6^. The corresponding symmetry axis passes through the middle of the tetramer near residue 191, in a direction perpendicular to the figure plane in Fig. 3A, upper part. Further constraints such as the maximal length of the linkers, sterical clashes terms, etc. were also used (see Methods). As a result, a new ‘compact’ tetramer model was obtained, which includes ‘backfolding’ of both coil1A and coil2 onto the coil1B tetramer. Specifically, the flexing at the linker L12 is close to 180°, whereby the coil2 segments align with the coil1B segments. The coil1A segment locates at an angle of ~ 45° relative to coil1B (Fig. 3B). Interestingly, coil1A could not be placed parallel to coil1B or in-between coil1B and coil2. The final compact model (Fig. 3B) satisfies 21 XLs out of 23 (Table 2). Compared to the elongated model (Fig. 3A), it additionally respects one XL between coil1A and coil1B (K120–K168) and three XLs between coil1B and coil2 (K188–K334, K223–K294, K223–K313), all four newly satisfied XLs being intradimeric.

One of the two violated XLs, K97–K402, is also intradimeric, connecting residues which are both close to the opposite ends of the rod domain (positions 93 and 404 respectively). As seen in Fig. 3B, additional mobility of the coil1A segment within the compact tetramer could in principle bring residues 97 and 402 sufficiently close for cross-linking. Of note, coil1A is known to form a CC dimer that is only marginally stable^30^. Accordingly, separation of the coil1A into two separate helices or even larger structural changes upon tetramer formation cannot be excluded, possibly enabling this XL. The second violated XL (K294–K402) is, in contrast, interdimeric. These two residues are located close to the opposite ends of coil2. Correspondingly, this XL would be respected through antiparallel arrangement of two dimers bringing their coil2 domains in approximate register. This situation corresponds to the A_22_ type assembly suggested by Steinert and colleagues^23^.

As a final step, we modelled the terminal domains onto both of our alternative tetramer models (the elongated and the compact) using the XLs found for these domains (Table 3). Here, no information was used as to whether a particular XL is intra- and interdimeric. Correspondingly, our modelling of the terminal domain simply aimed at satisfying as many XLs as possible, either within a dimer or across the dimers. The starting conformation for the head and tail domains was extracted from our recently published model of vimentin dimer obtained using the AlphaFold program^31^. In the context of a vimentin dimer, the head domains were predicted to ‘fold back’ on the N-terminal part of the rod domain (coil1A and half of coil1B), while forming little secondary structure beyond a random coil (Fig. 3C). In contrast, AlphaFold prediction suggests that the C-terminal part of the tail contains a β-hairpin motif (residues 440 to 464), and that two such motifs dimerize to form a continuous β-sheet (Fig. 3C).

In the elongated A_11_ tetramer model, the distal end of coil2 is located at a distance from the central part formed by the antiparallel coil1B domains. Correspondingly, we could simply attach the AlphaFold-modelled tail domains to each coil2 dimer. We found 10 XLs that lie entirely within the tail region, mostly also within the β-structural motif. All of these XLs are satisfied by the AlphaFold model. Of particular interest is the K445-K445 XL, situated at the symmetry axis of the β–structural dimer. Further XLs are made between the tail and coil2. Altogether, the predicted structure satisfied 19 of 29 (66%) of all XLs involving the tail.

The head domains were detected to make as many as 42 XLs to various parts of the rod (coil1A, coil1B or coil2), but almost no XLs fully within the head domain were detected (Table 3). When adding the head domains, we kept one of them (labelled ‘Head1’ in Fig. 3D) largely in the same conformation as predicted in the dimer context (Fig. 3C), which satisfies many XLs with coil1A and coil2 within the same dimer. However, most XLs between the head and coil2 could not be explained this way. Accordingly, the second head domain was semi-quantitatively modelled in a more compact way in the proximity of the N-terminal part of coil2 from the other dimer (‘Head 2’, Fig. 3D). Altogether, 75% of all XLs involving the head could be explained by the current model. Of note, five XLs were detected between the head and the tail domains (Table 3). These XLs could not be explained by the current ‘static’ model, but they are probably due to further conformational variability of both terminal domains.

A similar procedure was used to add the head and tail domains to the compact tetramer model (Fig. 3E). Here, both head domains were modelled in a more compact way (like ‘Head 2’ of the elongated model, Fig. 3D). The final model honors 77% of all XLs involving the terminal domains in the case of the elongated vimentin tetramer, and 79% for the compact tetramer.

### SEC-SAXS studies

**Figure 4:**
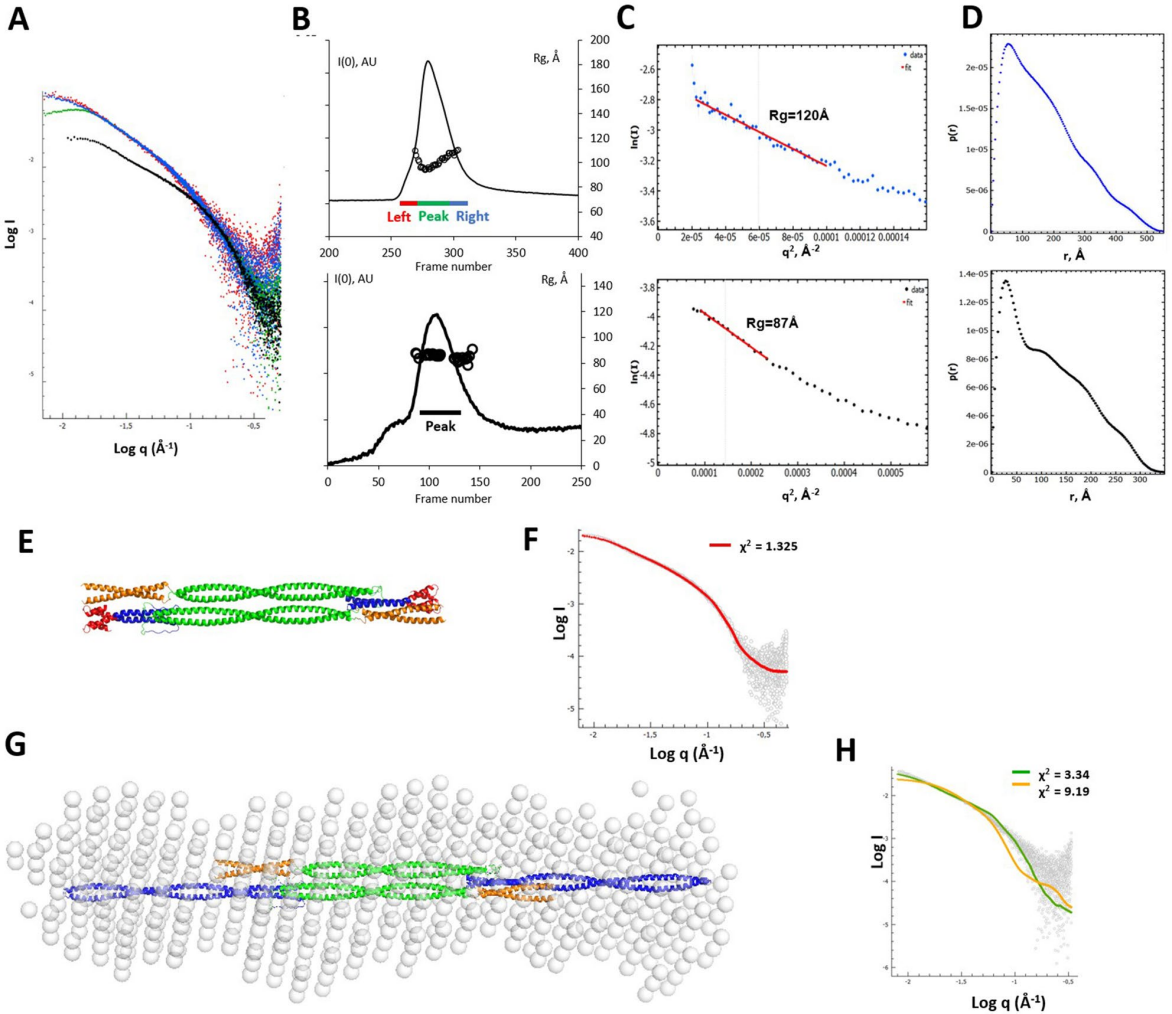
SEC-SAXS analysis. (**A**) SAXS curves for the FL vimentin corresponding to the beginning (red), center (green) and end (blue) of the elution. The SAXS curve for the elution peak of Vim93-302-Eb1 is overlaid in black. (**B**) Top: Elution profile of the FL vimentin tetramer from a Superose 6 Increase 3.2/300 column in 2 mM HEPES pH 8.2, 1% sucrose. The frames taken for the beginning, center and end of the elution peak are indicated below the curve. Across the elution peak, the R_g_ values per frame are plotted as black dots. Bottom: Elution profile for the Vim93-302-Eb1 fragment from a BioSEC-3 300 Å column in 10 mM HEPES buffer, 150 mM NaCl, pH 7.5. (**C**) Guinier analysis for the end of the elution peak of the FL vimentin (blue, top) and for the Vim93-302-Eb1 fragment (black, bottom). (**D**) The corresponding intraparticle distance distribution functions P(r). (**E**) Atomic model of Vim93-302-Eb1 shown as ribbon. The capping motif is colored red. (**F**) Scattering calculated from the Vim93-302Eb1 model (red line) fitted to experimental data for this fragment (open circles). (**G**) Low-resolution model of FL vimentin tetramer (semi-transparent spheres) obtained using SAXS data. The model is superimposed with the elongated vimentin tetramer model (ribbon). (**H**) Calculated scattering from the elongated tetramer model (green line) and compact tetramer model (orange line) fitted to experimental data for the end of SEC elution peak (open circles).

Next, we studied the vimentin tetramer using small angle X-ray scattering (SAXS) in solution. This method can provide low-resolution structural information towards a verification of the obtained atomic models. To this end, the FL vimentin tetramer was injected into a Superose 6 Increase 3.2/300 column in 2 mM HEPES pH 8.2, 1% sucrose, followed by inline SAXS measurements. The SAXS curves collected at the top of the elution peak revealed pronounced non-ideality (repulsion), which was apparent from a downward deviation of the lowest-angle signal (Fig. 4A, green curve)^32^. The same problem was noticeable from the plot of the SAXS-derived particle radius of gyration (R_g_) across the elution peak (Fig. 4B, black dots). Here, the apparent R_g_ value at the top of the peak was noticeably lower than for both the beginning and the end of the elution (‘smiling’ profile), reflecting an artefact caused by non-ideality. This complication was expected, given that a low ionic strength condition had to be used in order to avoid further assembly beyond tetramers. However, both the beginning (left-hand side) and the end (right-hand side) of the elution peak, which corresponded to smaller protein concentrations, are not significantly affected by non-ideality, according to the Guinier plot (Fig. 4C). Furthermore, the beginning and the end of the elution peak produce virtually identical scattering profiles (Fig. 4A, red and blue curves). This observation confirmed that the whole SEC peak corresponded to a structurally homogenous population of tetramers. Towards further processing, the SAXS curve collected for the end of the elution peak was used (see Methods).

In addition, we collected SEC-SAXS data for a vimentin rod fragment named Vim93-302-Eb1. This fragment was previously designed towards crystallographic studies and included vimentin residues 93 to 302, *i.e.* the rod domain except for the last ~ 100 residues of coil2. The fragment was additionally stabilized by the C-terminal capping motif Eb1, which we successfully used previously to produce crystallizable lamin fragments^33^. Although no crystals could be grown for Vim93-302-Eb1, this fragment could serve as a convenient reference sample for SAXS studies. Our SEC-MALS analysis indicated that this fragment was predominantly a tetramer in HS conditions (data not shown). Next, we performed a SEC-SAXS run under the same conditions (Fig. 4B). In contrast to the FL vimentin, this sample did not show any noticeable non-ideality, which was logical given the presence of 150 mM salt. Comparison of the SAXS curves, Guinier plots and intraparticle distance distributions for FT vimentin and the Vim93-302-Eb1 fragment is shown in Fig. 4A,C,D. Compared to Vim93-302-Eb1 tetramers, FL vimentin reveal considerably larger values for R_g_ (120 vs. 87 Å) and the maximal particle dimension D_max_ (~ 530 vs*.* ~ 340 Å), while the intraparticle distance distributions P(r) for both samples suggest a highly elongated, rod-like particle.

We assumed that the Vim93-302-Eb1 tetramer had the same three-dimensional structure as the middle part of the elongated FL vimentin tetramer model (Fig. 3C). The corresponding atomic model obtained after adding the small capping motifs at C-termini of both constituent dimers is shown in Fig. 4E. Indeed, a calculated theoretical SAXS curve for this model produces an excellent fit to the experimental data with χ^2^ = 1.32 (Fig. 4F), which indicates that our atomic model is in full agreement with the collected in solution SAXS data.

For the FL vimentin, we have used the recorded SAXS curve towards an ab initio low resolution shape reconstruction using the DAMMIF/DAMAVER program from the ATSAS software package. The obtained shape corresponds to an elongated, fairly uniform and straight particle (Fig. 4G). In line with this, analysis using the BODIES program suggested that the recorded SAXS data are optimally matched by a cylindrical particle with a diameter of 76 Å and a length of 490 Å (which is slightly below the maximal dimension D_max_ = 530 Å of the calculated P(r) function, Fig. 4D). In comparison, our elongated tetramer model (Fig. 3A) has a length of 540 Å. However, the maximal dimension of the cross-section of this model, in the absence of the terminal domains, is considerably lower (30–40 Å) than suggested through both DAMMIF and BODIES modelling.

Next, we calculated the fits between theoretical scattering from both our elongated and compact models of the vimentin tetramer (Figs. 3D,E) and experimental SAXS curves. The fit for the elongated model, while not perfect, is distinctly better (χ^2^ = 3.34) than the one produced by the compact model (χ^2^ = 9.12) (Figs. 4F,H). Finally, using the FoXS web server^34^ we obtained a slightly better fit (χ^2^ = 2.57), compared to the fit produced by the elongated model alone, when assuming a mixture of both elongated and compact tetramers. Here, optimal weight fractions were 0.64 for the elongated model and 0.36 for the compact model.

## Discussion

### Stabilization of vimentin tetramers in solution

Early on, it was established that cytoplasmic IF proteins including vimentin can be maintained in a soluble form in low ionic strength buffers, but they are capable of self-assembly into filaments upon salt addition^6^. The soluble species of FL vimentin is a tetramer. Indeed, using SEC-MALS measurements in 2 mM HEPES buffer, pH 8.2 (LS conditions) we could show that a recombinant FL vimentin sample has a constant molecular mass exactly matching the tetramer all across the SEC elution peak (Fig. 1A). Moreover, the same SAXS profile is recorded at the beginning and the end of elution in SEC-SAXS experiments (Fig. 4), pointing to a structurally homogenous population of tetramers under these conditions.

In this study, we wanted to explore which parts of the vimentin molecule are responsible for maintaining vimentin in the tetrameric form. In the past, assembly properties of several truncation variants of vimentin have been studied already. In particular, deletion of the head domain, but not of the tail domain, was shown to completely abolish in vitro filament assembly^10,24^. Moreover, DST cross-linking of the headless (Δ80) vimentin in 2 mM sodium phosphate pH 7.5 showed an intense dimeric band on the gel, while tetramer formation was observed upon 150 mM NaCl addition (HS conditions)^9^. Next, the published analytical centrifugation data suggest that vimentin rod domain was dimeric in LS conditions, but assembled into tetramers and a minor fraction of higher oligomers upon 150 mM NaCl addition^9^.

Our data support the importance of the head domain towards stabilization of the tetramers. Here, cross-linking of the isolated rod suggested only a minor presence of tetramers in LS conditions, compared to a major tetramer band in HS conditions, with all other parameters such as protein concentration and cross-linker excess being equal (Fig. 2B). It should be noted that the head domain is very basic (pI = 11.7) due to the presence of eleven arginine residues and no other charged residues, while the rod domain is acidic (pI = 4.7) all along its length (exactly the same pI is calculated for coil1B alone). It is therefore predictable that tetramerization of the isolated rod is compromised under LS conditions where repelling charges on the CCs are not shielded by the solvent.

Moving on to the HS conditions which approximate the physiological situation, we discovered that here the ΔNrod fragment lacking coil1A included only a minor fraction of tetramers in SEC-MALS experiments, while the rod was 100% tetrameric (Fig. 1B). Moreover, in cross-linking experiments with ΔNrod, no tetrameric species could be detected (Fig. 2B, Table 1). In line with this, we show that a yet shorter fragment corresponding to coil1B (residues 150 to 249) only forms dimers in solution in HS condition judging by both cross-linking and SEC-MALS, where we observed a constant dimeric mass at the elution peak. Previously, a similar fragment (residues 144–251) was reported to have a varied MALS-based molecular mass across the elution peak, roughly corresponding to a tetramer in 100 mM Tris–HCl buffer, 100 mM NaCl, pH 7.5^27^. However, this vimentin fragment contained an uncleaved affinity purification tag, which might have affected oligomerization. In fact, a recent SEC-MALS study of an isolated coil1B fragment of human lamin A also points to a pure dimer (Giel Stalmans, unpublished data). These consistent observations in solution are in stark contrast with the available crystal structures of coil1B constructs of vimentin, lamin A and K1/K10 which all reveal tetrameric assemblies of A_11_ type^35^. Apparently, the huge effective protein concentration in the crystals (hundreds of mg/mL) shifts the equilibrium towards tetramers.

In summary, our truncation experiments reveal that the presence of the coil1A domain is indispensable to stabilize the A_11_ tetramers under physiological conditions. This novel conclusion correlates with our chemical cross-linking data for FL vimentin tetramers (Table 2), just like earlier cross-linking studies^20,23^, which point to important interactions between the coil1A and coil2 regions of the two dimers. Moreover, these interactions are retained in our ‘mini-rod’ fragment Vim93-302-Eb1, which we confirm to form stable tetramers (Fig. 4E). Moreover, both coil1A and the beginning of coil2 form rather weak coiled coils, as we have recently studied in detail^31^. It is therefore feasible that these regions could undergo major structural changes upon mutual interactions during the tetramer formation. However, more experimental evidence would be needed before these phenomena could be incorporated into structural models, and our current models of the tetramer still assume the interaction of intact CC segments as rigid bodies (Figs. 3A,B). Finally, very recent cryoelectron tomography data on vimentin filaments supports a crucial role of both heads and coil1A segments in the filament architecture^18^.

### Use of cross-linking data towards modelling the tetramer

Historically, chemical cross-linking by Steinert and colleagues provided important data on IF assembly. In particular, these authors employed DST to cross-link vimentin at 0.5 mg/mL in 10 mM triethanolamine-HCl pH 8.0, without salt or in the presence of 10 or 150 mM KCl^23^. After proteolytic cleavage, the cross-linked peptides were isolated using liquid chromatography and identified through chemical sequencing. In total, eleven obtained XLs within the rod domain (some appearing only in the presence of salt) were interpreted as interdimeric. As a result, three distinct modes of lateral dimer alignment, A_11_, A_22_ and A_12_, were proposed under the assumption of straight, rigid rod-like dimers (Supplementary Table 3). Specifically, in the ‘tetramer conditions’ *i.e.* 10 mM triethanolamine-HCl pH 8.0 without salt, seven XLs were detected, of which five were assigned to the A_11_ mode and two to the A_22_ mode. Four further XLs, including two for the A_12_ mode were only observed upon salt addition.

In comparison, here we used a somewhat different low-salt buffer (2 mM HEPES, pH 8.2) for which we directly verified the presence of a homogenous population of vimentin tetramers. Thanks to the use of a superior cross-linking technology, we could identify 23 XLs (> 20 residues apart) within the rod domain, which is three times more than reported previously under similar conditions. Our data confirm all five A_11_ mode XLs of Steinert et al., as well as one of the two XLs that these authors assigned to the A_22_ mode (Supplementary Table 3).

An important asset of our approach was the use of isotope labelling, which helped us to unambiguously assign the XLs as being either inter- or intradimeric, enabling stricter restraints towards three-dimensional modelling. Specifically, of 23 XLs within the rod domain, 17 were interdimeric. Of those, we could explain 16 by creating an elongated A_11_ tetramer model (Fig. 3A). The last, single XL (K294–K402) could be explained by the presence of some type A_22_ tetramers, in addition to the dominant A_11_ tetramers. However, the same XL could be explained by other causes, such as a small degree of higher-order association of A_11_ tetramers. Importantly, none of our interdimeric XLs are compatible with either the remaining lateral mode A_12_ or longitudinal association of the dimers (A_CN_ mode). Our observations are therefore in agreement with the conclusions of Steinert and colleagues who postulated that the A_12_ or A_CN_ modes only take place in assembled filaments^23^.

Our final elongated A_11_ tetramer model includes ‘z-folding’ of linkers L12, which effectively reduces the total length of the tetramer. Moreover, we hypothesize that in solution this model could be prone to considerable conformational dynamics. Indeed, the extreme case of ‘backfolding’ of both coil1A and coil2 domains on the tetrameric core formed by the coil1B domains is represented by our compact A_11_ tetramer model (Fig. 3B). This model explains the four additional intradimeric XLs observed (Table 2).

### Complete atomic model of vimentin tetramer

Given the importance of the head domain for correct IF assembly as well as the reported role of the tail domain in controlling the filament width^24^, it is of great interest to produce a complete model of the soluble tetramer *i.e.* including the terminal domains. This is a challenging task since both domains are predicted to be very flexible and contain little secondary structure. In the past, it was argued that the interaction between the head and the CC parts should be driven by electrostatic attraction between the oppositely charged head and rod domains^4^. In line with this, we identified as many as 42 XLs between the head and the entire rod domain. Multiple residues all along the head domain were seen in the proximity of coil1A and coil1B, while some XLs even extend to coil2 (Table 3). These observations correlate with published site-directed spin labeling and electron paramagnetic resonance data which suggested the interaction of the first 32 head residues with other parts of the molecule^36^.

Furthermore, we could identify ten XLs within the vimentin tail domain, with especially K445 and K439 involved in multiple close-by interactions (Table 3). These findings align well with our prediction of a dimeric β-structural motif at the C-terminus of the tail domain (Fig. 3C). Next, we identified twelve XLs between the tail and the rod, with a majority of the contacts made by coil2. Interestingly, we also found five head-to-tail XLs, despite these domains attaching to opposite ends of the rod.

Based on SEC-SAXS analysis for the FL vimentin, we conclude that our elongated tetramer model (Fig. 3D) provides a reasonable approximation of the true conformation in solution. The first possible cause of the remaining discrepancy between the theoretical and experimental SAXS curves could be the variation in the conformation of the tetrameric core formed by the α-helical segments. Here, our compact tetramer model (Fig. 3E) appears to correspond to the extreme, most tightly folded case. Secondly, it should be expected that large portions of both the head and the tail remain poorly structured in the tetramer. It is clear that the obtained cross-linking restraints for the head and tail domains (Table 3) are not sufficient to model these flexible regions at atomic precision. Correspondingly, the current model presented in Fig. 3D should be considered just one possibility out of a multitude of conformations possible in solution. Such variable conformations of the terminal domains could in part explain the larger thickness of the rod-like particle suggested from ab initio SAXS-based modelling compared to our elongated atomic model (Fig. 4G). In contrast, the Vim93-302-Eb1 tetramer which did not include any larger flexible domains produced an excellent fit between the atomic model and SAXS data (Fig. 4F).

Of note, accuracy of the obtained molecular model of the FL vimentin tetramer varies considerably across its different parts. The core of the tetramer formed by two antiparallel coil1B dimers corresponds to a crystal structure (PDB code 3UF1, determined to 2.8 Å resolution) and therefore has atomic precision. The relative position of the coil1A and coil2 domains is defined by multiple cross-links (that are satisfied in both elongated and compact models) and thus accurate within the length of the cross-linkers used (3–4 nm). Finally, the relative position of coil1A and coil2 with respect to the coil1B tetrameric overlap can vary drastically, with the elongated and compact models representing the two extremes as discussed. The terminal domains also remain highly flexible under the studied conditions.

## Conclusions

Here we provided a detailed structural characterization of soluble vimentin tetramers which are based on the A_11_ alignment of elementary dimers. In particular, we conclude that the coil1B domain alone is not capable of tetramerization in solution. At the same time, tetramers made by two antiparallel, aligned coil1B dimers were observed under the extreme conditions of crystallization in the past, and the obtained atomic structures^27,35,37^ adequately reflect the interdimeric interactions within the FL tetramer, as we show here using chemical cross-linking. In summary, both the head domain (as reported previously) and coil1A domains (this work) make crucial contributions to the stabilization of soluble FL tetramers. Moreover, we demonstrate that in low ionic strength conditions the tetrameric species is not a tightly folded structure but retains a considerable flexibility, including ‘backfolding’ of both coil1A and coil2 domains on the central core made by the coil1B tetramer, and a yet higher conformational variability of the terminal domains. For the future, especially the conformation of the head domain deserves further exploration, given its necessary involvement in filament assembly upon ionic strength increase. Here, experimenting with gradual head truncations as well as cross-linking upon replacement of selected head arginines with more reactive lysines^4^ appears especially promising.

## Materials and methods

### Expression and purification of full-length vimentin

FL vimentin was overexpressed in *E. coli* using Rosetta 2 (DE3) pLysS cells (Merck, Germany) and a pETCH vector following already optimized protocols^38^. The expression construct was a generous gift from Prof. Harald Herrmann. Single cell colonies were grown overnight on Luria–Bertani (LB) plates containing 100 μg/mL ampicillin and 10 μg/mL chloramphenicol. One colony was pre-cultured for 7 h at 37 °C in 2 mL of LB medium supplemented with antibiotics. For inoculation, 1 mL of the pre-culture was added to 2L of ZYP-5052 medium and incubated at 37 °C for 24 h. This time was sufficient for the cell culture to reach an OD_600_ of 9–10.

Next, the target protein was purified from inclusion bodies. To this end, the cells were harvested by centrifugation at 6 k RCF for 8 min at 4 °C and stored at − 80 °C. Approximately 6 g of cell pellet were re-suspended in 70 mL lysis buffer (20 mM Tris–HCl pH 7.5, 10 mM MgCl_2_, 0.1% Triton X-100, 5% Sigmafast inhibitor cocktail (Sigma-Aldrich, Overijse, Belgium and 100U Cryonase (cold-active nuclease (Takara Bio Europe SAS, Saint-Germain-en-Laye, France)), sonicated (70% amplitude, total 1 min of action, 1 s ON/1 s OFF) and centrifuged at 20 k RCF at 4 °C, (Sigma 6K15, Sigma, Osterode am Harz, Germany). The pellet was subjected to a wash procedure that included subsequent cycles of by-hand homogenization (15 strokes) in a glass Potter–Elvehjem tissue grinder and centrifugation at 20 k RCF for 30 min at 4 °C. The solutions were used in the following order: wash buffer (lysis buffer with 0.5% Triton X-100); high-salt buffer (lysis buffer with 1.5 M NaCl); lysis buffer; 10 mM Tris–HCl pH 7.5. After the wash the pellet was resuspended in ion-exchange buffer A (IE-A; 8 M urea, 5 mM Tris–HCl, 2 mM DTT, 5 mM methylamine hydrochloride (MAC), pH 7.5) and purified using 2 × 5 mL HiTrap Q HP columns (GE Healthcare Europe GmbH, Diegem, Belgium) with a linear NaCl (0–0.3 M) gradient elution. The peak fractions were pooled and diluted 5 times with the IE-A buffer. Then, the diluted fractions were loaded on 2 × 5 mL HiTrap SP HP columns (GE Healthcare Europe GmbH, Diegem, Belgium) in IE-A buffer and eluted with a linear NaCl (0–0.3 M) gradient. Thereafter, the samples were dialyzed into the dimer buffer (6.2 M urea, 5 mM Tris–HCl, 1 mM MAC, 0.3 mM DTT, pH 8.4), applied on 1 × 1 mL HiTrap Q HP column (GE Healthcare Europe GmbH, Diegem, Belgium) and eluted with a linear guanidinium-HCl gradient (0–0.3 M). The collected fractions were stored individually at − 80 °C. All proteins were purified to single band purity on Coomassie (“blue silver”) stained SDS-PAGE in mg amounts and stored at − 80 °C. For further experiments, the urea was removed by a step-wise dialysis using 2 mM HEPES buffer (pH 8.2) with 4 M, 2 M and finally 0 M urea^39^.

### Expression and purification of ^15^N-labeled vimentin

Expression of ^15^N labeled protein was performed in Rosetta 2 (DE3) pLysS cells (Merck, Germany). To this end, P-0.5G and ^15^N-5052 media were used^40^, containing ammonium chloride and ^15^N labeled ammonium chloride as nitrogen source respectively. The pre-culture was prepared as described above. To inoculate 200 mL of the P-0.5G medium, 2 mL of the pre-culture was used. The culture was incubated at 28 °C overnight in a 250 mL sterile disposable PETG flask (Thermo Scientific, USA) till OD_600_ 2.9 was reached. Afterwards, 50 mL of the P-0.5G culture was spun down into a conical tube for 20 min at 4 k rpm. The pellet was resuspended in 1 mL of ^15^N-5052 medium and transferred into 100 mL of ^15^N-5052 culture in a 250 mL sterile flask. The culture was incubated for 24 h at 37 °C to reach the OD_600_ of 4. ^15^N-labeled protein was purified as described above.

### Expression and purification of the Vim93-302-Eb1 construct

A construct containing vimentin residues 93 to 302 followed by the Eb1 capping motif^31,33^ was expressed using the pETSUK2 vector^41^. The N-terminal residue of the capping motif was chosen to preserve a continuous heptad periodicity from the vimentin fragment into the cap. The sequence was synthesized as a gBlocks Gene Fragment (IDT DNA Technologies) and inserted into the vector using ligation-independent cloning^41^. The construct was overexpressed in Rosetta 2 (DE3) pLysS strain (Merck, Germany) using auto-induction ZYP-5052 media to an OD_600_ of 10, and the cells were harvested via centrifugation. Purification was carried out in non-denaturing conditions as described in^33^.

### SEC-MALS

Static multiangle light scattering was measured using a Dawn Heleos device (Wyatt, Santa Barbara, USA) and the results were analyzed with the ASTRA 5.3.4 software (Wyatt). 100µL of the protein sample were injected into a Superdex 200 Increase 10/300 GL column, pre-equilibrated with the required buffer, and eluted at 0.5 mL/min. The injected sample concentrations were 0.40 mg/mL for FL vimentin in LS buffer, and 0.79 mg/mL for the rod construct, 0.55 mg/mL for the ΔNrod and 1.52 mg/mL for the coil1B construct (all in HS buffer).

### Chemical cross-linking and MS analysis

Cross-linking experiments were carried out as described in^33^. FL vimentin (0.15 mg/mL in 2 mM HEPES buffer, pH 8.2) was subjected to cross-linking with disuccinimidyl glutarate (H_6_-DSG/D_6_-DSG, molar ratio 1:1, Creative Molecules, Victoria, Canada), disuccinimidyl dipropionic urea (DSPU, CF Plus Chemicals, Brno-Řečkovice, Czech Republic) and disuccinimidyl dibutyric urea (DSBU, CF Plus Chemicals). Note that throughout this manuscript H_6_-DSG/D_6_-DSG was labelled simply as DSG. Truncated vimentin fragments at 0.4 mg/mL were cross-linked with DSG and DSBU in either 2 mM HEPES, pH 8.2, or in 10 mM HEPES, 150 mM NaCl, pH 7.5.

After digestion with trypsin at 37 °C for 2 h (enzyme:protein ratio of 1:10), the samples were fractionated on a reversed-phase column using an Agilent UHPLC system and analyzed using two different MS/MS devices. First, trapped ion mobility spectrometry coupled to time-of-flight (timsTOF) measurements were performed using a timsTOF Pro device (Bruker Daltonics, Bremen, Germany) as described in^42^. In addition, samples that had been cross-linked with DSPU were additionally subjected to Fourier transform ion cyclotron resonance (FT-ICR) measurements on an 15 T solariX XR device (Bruker Daltonics, Bremen, Germany) essentially as described in^33^. All experiments were performed in triplicate.

For the timsTOF data, the Merox 2.0.1.4 software was used^43^. The following settings were applied: cleavage at C-end of Lys and Arg with a maximum of 3 missed proteolytic cleavages and blocked proteolysis by Pro, fixed carbamidomethylation of cysteines and variable oxidation of methionines. Cross-linker specificity was set for N-termini, Lys, Ser, Thr and Tyr for homobifunctional cross-linkers. Error tolerance was set to 10.0 ppm for parent ions and 50.0 ppm for fragment spectra. Minimum charge of the precursor was set to + 2. Minimum number of fragments per peptide was equal to 3 and minimum signal-to-noise ratio was set to 1.5. Scoring settings were as follows: prescore of at least 10% intensity, false-discovery rate (FDR) of 1.0% and score cut-off of more than 50.0. All cross-linked positions were manually checked.

Processing of raw FT-ICR data was done by the Data Analysis 4.4 software (Bruker Daltonics) and exported to the mascot generic files (mgf) using the SNAP 2.0 algorithm. The mgf files were analysed with MeroX 1.6.0.1^44^ for the MS-cleavable cross-linker DSPU. Search setup was as follows: cleavage at C-end of Lys, Tyr and Arg with a maximum of 5 missed proteolytic cleavages and blocked proteolysis by Pro, minimum and maximum peptide length set for 3 and 15 respectively, fixed carbamidomethylation of cysteines and variable oxidation of methionines. Cross-linker specificity was set for N-termini, Lys, Ser, Thr and Tyr. Dead-end and intrapeptidal XLs were considered during the analysis. MS error tolerance was set to 1.0 ppm for precursors and 2.0 ppm set as fragment ion precision. Mass limit from 200 to 6000 Da and minimum signal-to-noise ratio was set to 1.0. No prescoring nor FDR cut-off filters were applied. All MeroX outputs with a score higher than 7 were manually checked. At least one b or y ion per peptide was required, in this way confirming the MeroX proposed peptide sequence, as recommended in^45^. Moreover, the presence of reporter ions for both peptides was mandatory, with minimum 3 out of 4 are allowed considering the high mass precision of the MS instrument. Maximum 2 neutral losses per ion were allowed for all cross-linking data. Finally, the manually checked XLs were mass ranked to exclude possible monolinked peptides and dead-end XLs.

### ^14^N/^15^N cross-linking and data processing

Vimentin ^14^N (L, light) and vimentin ^15^N (H, heavy) samples were each prepared separately in the dimer buffer (6.2 M urea, 5 mM Tris–HCl, 1 mM MAC, 0.3 mM DTT, pH 8.4). Subsequently, the concentration of dimeric samples was determined by UV spectroscopy (A_280_). Thereafter the ^14^N and ^15^N samples were mixed in 1:1 molar ratio and stepwise dialyzed to decrease urea concentration, ultimately forming tetramers in 2 mM HEPES buffer (pH 8.2). The sample was cross-linked at a concentration of 0.3 mg/mL with DSG, DSS, DSPU and DSBU cross-linkers in 50 × molar excess. After trypsinolysis, the samples were measured by LC–MS (Agilent 1290 and 15 T solariX XR FT-ICR), obtaining high-resolution data suitable for data processing by in-house developed software LinX^25^.

The uniqueness of the LinX program is the ability to assign and calculate the ratio of light/heavy (L/H) cross-linked peptides, reflecting their origin. Based on quantification of the L/H ratio, XLs can be used for modeling and determination of interactions sides responsible for tetramer assembly. A schematic representation of possible dimeric interactions when cross-linking ^14^N/^15^N tetramers, after cross-linking and proteolytic digestion, is shown in Supplementary Fig. 2B.

The data obtained from the LC–MS analysis were processed using the Data Analysis 5.0 program equipped with the SNAP algorithm, ensuring the correct assignment of the charge states of the peptides and their conversion to monoisotopic masses. The data processed in this way were exported and further processed using the LinX 1.1 program, enabling the assignment and quantification of cross-linked peptides labeled with ^14^N and ^15^N nitrogen. In order to create a theoretical library of possible peptide connections and search the data from this experiment, appropriate parameters were set in the LinX program. Firstly, the amino acid sequence of vimentin was inserted in the FASTA format, then the used protease was chosen, i.e. trypsin, which specifically cleaves the peptide bond after the amino acids arginine and lysine, unless they are followed by proline. The maximum number of omitted cleavage sites was set to three. Then a variable modification corresponding to, oxidation of methionine and a fixed modification corresponding to carbamidomethylation of cysteine were set. Furthermore, the cross-linking agent used and its specificity towards lysine and the N-terminus were defined. The measurement error was set to a maximum of 2 ppm. Finally, the obtained data, i.e. the experimental *m/z* values of detected peptides, were exported to the program. The LinX program was used to compare the experimentally determined *m/z* values with the theoretical mass values of the cross-linked peptides, which can be formed by cleavage of vimentin with trypsin after cross-linking. In case the relative deviation of the experimental and theoretical values did not exceed 2 ppm, the experimental values were assigned to specific cross-linked peptides. Cross-linked or modified peptides identified by the LinX program were manually traced in the mass spectra using the Data Analysis 5.0 program. Based on the information obtained using the LinX program, interdimer and intradimer XLs were quantified. To this end, the α-ratio was calculated as

$$\frac{{\sum {I_{{{{^{14} {\text{N}}} \mathord{\left/ {\vphantom {{^{14} {\text{N}}} {^{14} {\text{N}}}}} \right. \kern-0pt} {^{15} {\text{N}}}}}} + } I_{{{{^{15} {\text{N}}} \mathord{\left/ {\vphantom {{^{15} {\text{N}}} {^{14} {\text{N}}}}} \right. \kern-0pt} {^{14} {\text{N}}}}}} }}{{\sum {I_{{{{^{14} {\text{N}}} \mathord{\left/ {\vphantom {{^{14} {\text{N}}} {^{14} {\text{N}}}}} \right. \kern-0pt} {^{14} {\text{N}}}}}} + } I_{{{{^{15} {\text{N}}} \mathord{\left/ {\vphantom {{^{15} {\text{N}}} {^{15} {\text{N}}}}} \right. \kern-0pt} {^{15} {\text{N}}}}}} }} \times 100$$

in which *I* expresses the intensity of the cross-linked peptides of ^14^N/^15^N, ^15^N/^14^N, ^14^N/^14^N and ^15^N/^15^N types. All contributions of the isotopic envelope intensities of given peptide form were included in the sum.

### Modelling

An atomic model of vimentin rod dimer was produced using our own CCFold algorithm^26^. Initially, two such dimers were aligned to match the antiparallel overlap of the coil1B domains as observed in the crystal structure of the vimentin coil1B fragment (PDB code 3UF1) which forms tetramers^27^*.*

To produce the elongated tetramer model, the positions of the coil2 dimers relative to the coil1B tetrameric overlap were optimized in PyMOL to match as many XLs as possible. The L12 linkers were then refined in Chimera using its MODELLER plugin. Finally, the model was energy minimized using GROMACS.

To produce the compact tetramer, we used the integrative modeling platform (IMP) v2.16.0^29^. The software allowed us to integrate different data sources as modeling constraints. The head and tail domains were not considered at this point. The IMP topology of the overall model specified five separate regions for each monomer: coil1A (residues 97–140), L1 (141–144), coil1B (145–250), L12 (251–265) and coil2 (266–404). Coil1A was treated as a rigid body within each dimer, forming two coil1A rigid bodies for the whole tetramer. Coil2 was treated in the same way. The tetramer formed by coil1B was treated as a single rigid body. The linkers L1 and L12 were configured as monomeric rigid bodies, leading to a total of eight rigid bodies. Given that the linkers are rather short, they effectively formed flexible hinges.

Three different types of constraints in the IMP were used. One was a connectivity constraint (distance 3.6 Å) between different rigid bodies with a weight of 1. To reduce steric clashes within the model, an excluded volume sphere constraint with a weight of 20 was used. Finally, cross-linking constraints between Cα-positions were used with a weight of 40 and distance cut-offs of 30 Å for DSG, 35 Å for DSS and DSPU, and 40 Å for DSBU.

Here, intradimeric XLs were configured to have two separate ambiguous sets (*i.e.* sets of restraints of which at least one had to be honored), one set for each dimer. Each set had four permutations of XLs, two intrachain XLs and two intradimeric. For instance, for a tetramer with chains A/B as one dimer and chains C/D as another dimer, one set contained A->A and B->B (intrachain) and A->B and B->A (intradimeric) XL constraints, while the other set contained C->C and D->D (intrachain) and C->D and D->C (intradimeric) constraints. Similarly, interdimeric XLs contained two separate ambiguous sets that mirrored each other. For the aforementioned tetramer, one set contained A->C, A->D, B->C and B->D interdimeric XLs. The other mirrored set contained C->A, C->B, D->A and D->B. This way the modelling was guided to produce a symmetric tetramer.

We then ran a Monte Carlo replica exchange simulation with IMP, configured to run in atomic resolution mode (resolution 0) until the model scores stabilized (between 2000 and 4000 iterations). We also used simulated annealing with a max temperature of 2.5 and min of 1.0. Finally, IMP was configured to do a random start, so that the starting model was randomly perturbed before beginning the run while honoring the topology restraints. This first iteration ran 12 separate modeling processes, each with random starts. The best scoring model was fed into GROMACS version 2021.4^46^ to do an energy minimization using AMBER99SB. The second iteration used the best scoring model from the previous iteration with the same constraints. We added an additional cylindrical constraint with a weight of 5, which imposed a penalty for atoms outside of a z-axis aligned cylinder with a radius of 32.5 Å. This had the effect of making the overall structure more compact without violating additional XLs. Again, several modeling processes were run in parallel and the best scoring model was energy minimized in GROMACS.

The third and final iteration used again the best scoring model from the previous iteration and the same set of constraints with two main differences. First, the cylindrical constraint was reconfigured to exclude coil1A to allow it to be positioned orthogonally to the z-axis if it would score better. Second, a soft symmetry constraint was added with a weight of 0.001 per atom. This constraint nudged the modeling process to be rotationally symmetrical around the Y-axis by penalizing atoms that deviated too much. This modeling process resulted in the best scoring model that was similar in overall structure to the one from the second iteration step but is much more symmetrical, without violating any crosslinks. This model was then further energy minimized.

At the final stage of modelling, head and tail domains were added. These domains were extracted from the model of the FL vimentin dimer which had been produced using the AlphaFold algorithm^47^ as described in^31^. The predictions have low confidence except for the small α-helical region around residues 10–15 in the head, and the cross-dimer beta sheet in the tail.

To visualize XLs in the final tetramer models, we used the PyXlinkViewer plugin for PyMOL^48^.

### SEC-SAXS

Data for the FL protein were collected at the bioSAXS beamline B21 at Diamond Light Source (Harwell, United Kingdom) using the mail-in service. Prior to the experiment, vimentin was dialyzed into 2 mM HEPES pH 8.2 supplemented with 1% sucrose. 60µL vimentin sample (2.8 mg/mL) was applied on a Superose 6 Increase 3.2/300 column (GE Healthcare) pre-equilibrated with 2 mM HEPES pH 8.2, 1% sucrose. This column is suitable for analysis of proteins with molecular weights up to 4MDa. The flow rate was set to 0.075 mL/min. The output flow from the Agilent 1200 HPLC was directed through an in-vacuum quartz capillary with a diameter of 1.5 mm. In total 599 frames were collected on an EigerX 4 M (Dectris, Switzerland) detector at a distance of 4.014 m from the sample.

For the Vim93-302-Eb1 construct, SEC-SAXS data were collected at the Synchrotron Soleil, beamline SWING (Saint-Aubin, France). Prior to the experiment, the fragment was precipitated with ammonium sulphate and redissolved in 10 mM Tris–HCl 150 mM NaCl pH 7.5. Next, 40μL of 7 mg/mL was injected into a pre-equilibrated Agilent Bio SEC-3 300 Å column (Agilent, Santa Clara, California, United States) by an automated sample changer at 15 °C. The flow rate was set to 0.3 mL/min. In total 840 frames were recorded using an AVIEX170170 CCD detector.

Data processing was performed with the ATSAS 3.0 package^49^. The CHROMIXS module was used to analyze the individual SAXS frames obtained upon SEC elution with respect to the radius of gyration (R_g_) values. Multiple frames for the beginning, middle, and the end of the elution peak for the FL vimentin were averaged and buffer-subtracted. For Vim93-302-Eb1, all frames of the peak were averaged towards further processing. Plotting of SAXS curves and Guinier analysis were done with PRIMUS. The distance distribution functions P(r) were calculated using GNOM. Fitting of the experimental scattering data and theoretical scattering curve calculated from atomic model was performed with CRYSOL^50^. Low-resolution ab initio models were calculated using the DAMMIF/DAMAVER and BODIES modules of ATSAS. Multi-state model fitting was performed using the FoXS web server^34^.

## Supplementary Information

Supplementary Figures.

Supplementary Tables.

## Data Availability

Final atomic models of vimentin tetramers as shown in Figs. 3D,E can be downloaded from https://gbiomed.kuleuven.be/english/research/50000715/50000719/vimentin_tetramer_models.
