# Author Correction: Molecular structure of soluble vimentin tetramers

**DOI:** 10.1038/s41598-024-79005-x

**Published:** 2024-11-20

**Authors:** Pieter-Jan Vermeire, Anastasia V. Lilina, Hani M. Hashim, Lada Dlabolová, Jan Fiala, Steven Beelen, Zdeněk Kukačka, Jeremy N. Harvey, Petr Novák, Sergei V. Strelkov

**Affiliations:** 1 Laboratory for Biocrystallography, KU Leuven, 3000 Leuven, Belgium; 2 Department of Chemistry, KU Leuven, 3000 Leuven, Belgium; 3 Department of Biochemistry, Charles University, 12800 Prague, Czech Republic; 4 Institute of Microbiology of the Czech Academy of Sciences, 14220 Prague, Czech Republic

Correction to: *Scientific Reports* 10.1038/s41598-023-34814-4, published online 31 May 2023

The original version of this Article contained an error in the Data Availability statement.

A non-functional URL link had been provided for accessing the atomic models of vimentin.

“Final atomic models of vimentin tetramers as shown in Figs. 3D, E can be downloaded from https://pharm.kuleuven.be/Biocrystallography/vimentin_tetramer_models.”

now reads:

“Final atomic models of vimentin tetramers as shown in Figs. 3D, E can be downloaded from https://gbiomed.kuleuven.be/english/research/50000715/50000719/vimentin_tetramer_models”

The original Article has been corrected.

