# Supplementary material for: Molecular structure of soluble vimentin tetramers
Source: Sci Rep. 2023 May 31;13:8841. doi: 10.1038/s41598-023-34814-4 (PMC10232555; doi:10.1038/s41598-023-34814-4)
Supplement: Supplementary file 1 — Supplementary Figures. [file 41598_2023_34814_MOESM1_ESM.docx]

**Supplementary Figures**


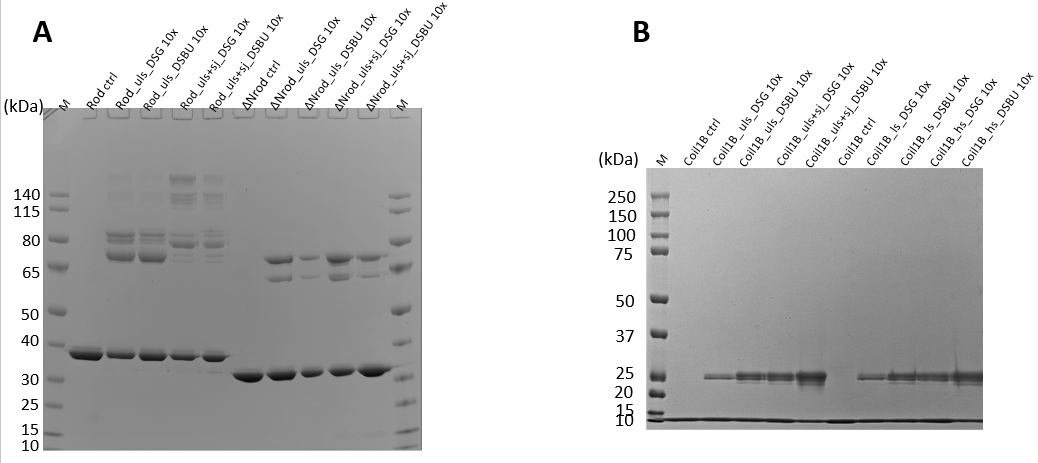

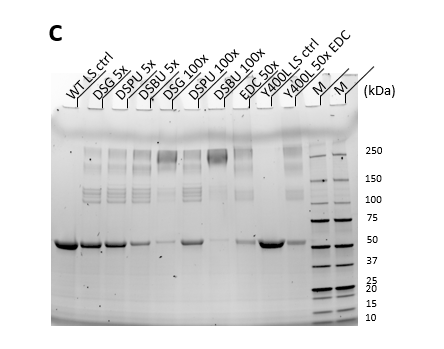


***Supplementary Figure 1.*** *Reducing SDS-PAGE analysis of chemically cross-linked vimentin samples (uncropped gels). Molar excess of the cross-linker over the protein is indicated. A. Vimentin rod and ΔNrod constructs cross-linked with DSG and DSBU in either LS or HS conditions. B. Coil1B construct. C. FL vimentin in LS buffer before (control, ctrl) and after chemical cross-linking with DSG, DSPU and DSBU.*

***
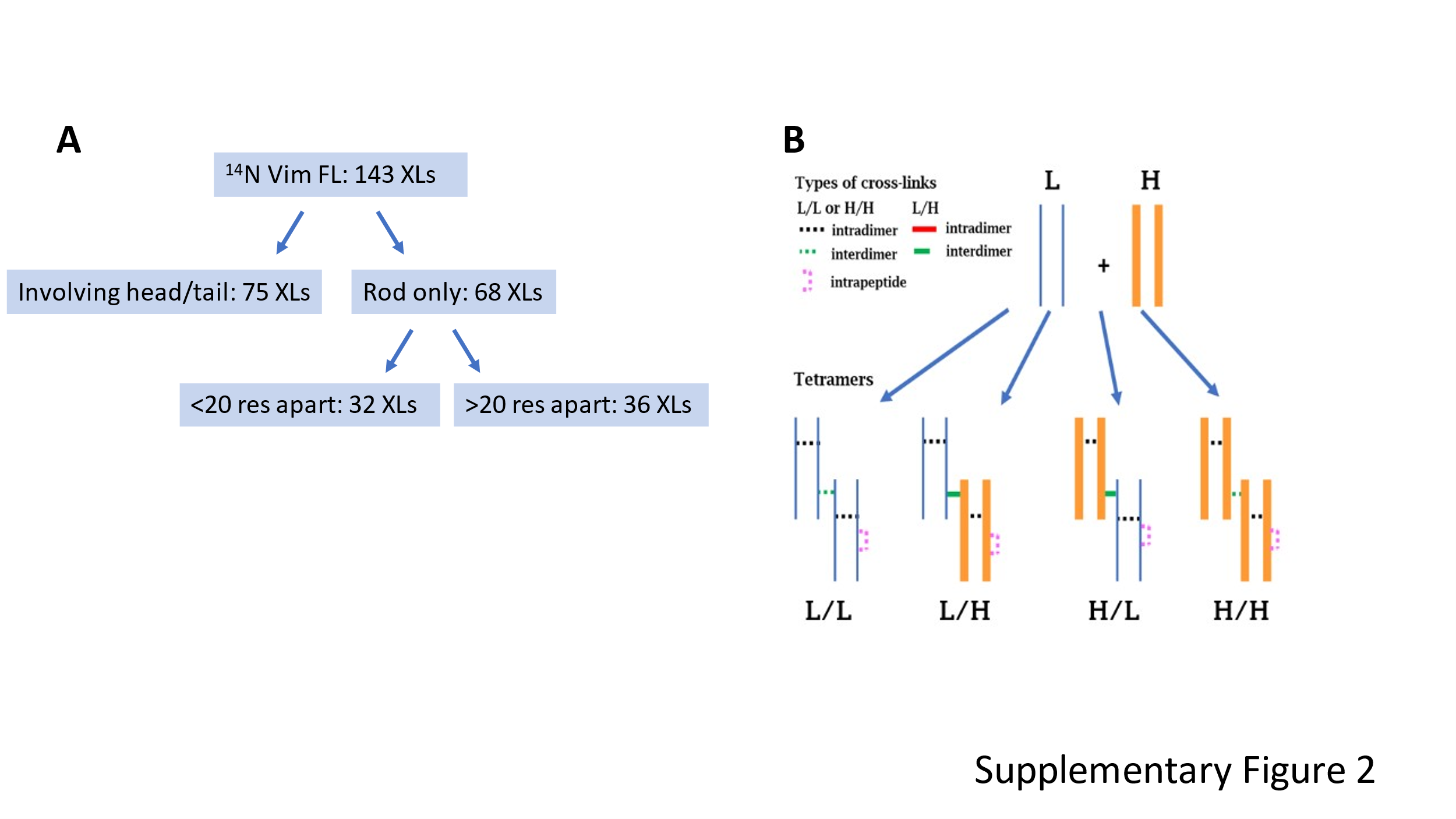
Supplementary Figure*** ***2.*** *MS data processing of cross-linked FL vimentin. A. Total number of XLs (in triplicate) identified for all cross-linkers in ^14^N tetramers as well as the number per subdivision. B. Cross-linking of ^14^N/^15^N vimentin tetramers produced through mixing stable dimers. Schematic representation of all possible intra- and interdimeric XLs.*
